# Interactive effect of dietary levels of calcium and 25-hydroxy vitamin D3 on the performance, serum biochemical concentration and digestibility of laying hens from 61 to 70 weeks of age

**DOI:** 10.5713/ab.22.0003

**Published:** 2022-05-02

**Authors:** Chun Ik Lim, Kyeong Seon Ryu

**Affiliations:** 1 Department of Animal Science, College of Agriculture and Life Sciences, Jeonbuk National University, Jeonju 54896, Korea

**Keywords:** 25-Hydroxy Vitamin D3, Calcium, Laying Hens, Nutrition Digestibility, Performance, Serum Concentration

## Abstract

**Objective:**

The present research was conducted to evaluate the interactive effect of dietary concentration of calcium (Ca) and 25-hydroxy vitamin D3 (25OHD3) on the performance, blood composition and digestibility of laying hens.

**Methods:**

A total of 540 Hy-line brown laying hens aged 61 to 70 weeks were randomly allotted in a 3×3 factorial arrangement, consisting of three levels of 25OHD3 (0, 25, and 50 μg/kg) and three levels of Ca (3.5%, 4.0%, and 4.5%). All diets had basal concentration of 3,000 IU/kg of vitamin D3 including the 2,800 kcal/kg of metabolic energy and 16% of crude protein.

**Results:**

The results showed that interactive effect (p<0.05) between Ca and 25OHD3 was such that dietary 25OHD3 linearly increased interleukin-6 at all levels of Ca inclusion. Interaction (p<0.05) occurred with the highest parathyroid hormone in laying hens that received dietary concentration of Ca (3.5%) with 25OHD3 (50 μg/kg), and Ca (4.0%) with 25OHD3 (50 μg/kg). Egg production and egg weight significantly (p<0.05) increased in the 4.5% Ca group compared to the 3.5% to 4.0% Ca groups. Egg shell thickness and tibia bone length also increased (p<0.05) in groups fed a high-Ca diet (4.0% to 4.5%). Phosphorus digestibility significantly (p<0.05) increased along with dietary Ca level. Among the tested 25OHD3 groups, higher (p<0.05) egg production and tibia thickness were present in hens fed 50 μg/kg of 25OHD3. Furthermore, Ca digestibility serum Ca and 25OHD3 were significantly increased in group offered 50 μg/kg of 25OHD3.

**Conclusion:**

The results gathered in this study indicate that dietary concentrations of 4.0% to 4.5% Ca and 50 μg/kg 25OHD3 improve the performance of hens from 61 to 70 weeks of age.

## INTRODUCTION

Calcium (Ca) and vitamin D3 (VD3) are considered critical nutrients for laying hens, and accordingly are regularly added to their diets. These nutrients play a significant role in eggshell formation and in maintaining skeletal integrity [1]. Poor bone and shell quality that results in lameness is responsible for a significant amount of economic loss to layer producers. The higher egg production rate of laying hens means that a large quantity of Ca is particularly necessary in their case [2]. Laying hens obtain Ca for eggs shell synthesis primarily from two sources; feed (the intestinal route) and by using reserves stored in the medullary bones [3]. Much of the Ca used by laying hens comes from limestone and oyster shells. The oyster shells provide a large amount of Ca that gradually releases the Ca, and act as reserve to be used when the hens are fasting [4].

Ca is used in many metabolic functions. The main functions of Ca are associated to the concentrations of P and VD3 [5]. Ca in the blood is used during eggshell synthesis, which stimulates the secretion of parathyroid hormone (PTH). This, in turn, promotes bone resorption to maintain the blood’s Ca level within a normal range. Notably, higher bone resorption rates at the laying stage are more likely to cause weak bones in the laying hens at the end of the production phase [6]. VD3 synthesis occurs in the skin by the catalytic action of ultraviolet light on 7-dihydrocholesterol. As commercial laying hens are mostly reared indoors where they receive insufficient sunlight to convert 7-dihydrocholesterol to VD3, they are prone to bone abnormalities [7]. Laying hens accordingly receive VD3 supplements to their diets to support their bone strength, egg production, and maintain appropriate blood Ca levels. After its absorption in the intestine VD3 is conveyed to the liver, where it is hydroxylated at position 25, resulting in 25-hydroxycholecalciferol (25OHD3) [8]. The resultant metabolite is transported to the kidneys and hydroxylated at carbon 1, giving rise to 1,25-dihydroxycholecalciferol (1,25(OH)2D3). Including different types of VD3 metabolites in a diet enhances vitamin D’s availability and effects [9]. Following molting, at the time of second production phase, laying hens restore their Ca levels to improve eggshell quality. This restoration mechanism operates completely independent of egg size, and thus the quality of the eggshells declines as the bird ages. Persistent Ca and VD3 misbalance results in higher rates of egg loss among commercial laying hens [10]. Ca and VD3 requirements must be updated to improve the egg quality and productivity for finishing stage of laying hens.

Thus, our study was designed to investigate the effect of dietary levels of Ca and 25OHD3 on the performance, serum biochemical concentration and digestibility of laying hens from 61 to 70 weeks of age to explore how laying hens respond to interaction of dietary supplementation between Ca and 25OHD3. Such investigations may provide evidence of whether dietary 25OHD3 supplementation exerts roles in finishing stage of laying hens following performance by improving Ca digestibility.

## MATERIALS AND METHODS

### Animals, managements and diets

The study was carried out at the Poultry Experimental Station of the Department of Animal Sciences at Jeonbuk National University in the Republic of Korea. The protocols for the experiment were approved by the Jeonbuk National University Institutional Animal Care and Use Committee (JBNU 2021-0175). A total of 540, 61-week-old, HyLine Brown laying hens were used and allotted in cages (2 birds/cage) measuring 1,200 cm^2^ (600 cm^2^/bird) in an experimental windowless house. A room temperature of 25°C±5°C and a photoperiod of 16/8 h light/dark cycle were maintained throughout the experimental period. A completely randomized experimental design in a 3×3 factorial arrangement, consisting of three levels of 25OHD3 (0, 25, and 50 μg/kg) and three levels of Ca (3.5%, 4.0%, and 4.5%) in a layer diet was used. Each group was distributed into five replicates, with twelve hens per replicate.

The vitamin sources were included in the diet to supply 3,000 IU/kg of VD3. The 25OHD3 (Hy-D) used in the vitamin premix was donated by DSM Nutritional Products Ayr, ON, Canada. All birds were fed with isoenergetic and isonitrogenous mash diets for 10 weeks. The diets were based on corn and soybean meal, formulated to meet the nutrient requirements of laying hens as recommended in the Korean Feeding Standard for Poultry [11]. The composition of the experimental diets is shown in Table 1. Feed and water were provided *ad libitum*.

### Sample processing and analyses

The fresh diets were feed every day. The feed intake (g/bird/d) and feed conversion ratio (FCR) were evaluated on a weekly basis by weighing the amount of feed offered in the beginning and at the end of this experiment.

Eggs were collected daily to determine the rate of eggs production. The mean egg weight was determined as the daily average weight of eggs, excluding abnormal eggs. After 6, 8, and 10 weeks five eggs from each replicate were collected, weighed individually, and stored overnight at room temperature for subsequent measurements. Eggshell thickness without the shell membrane was tested by micrometer (Digimatic micrometer, Series 293 330, Mitutoyo, Japan).

Blood samples were taken between 8:00 and 9:00 AM by puncturing the wing vein from hens of each replicate. These samples were subjected to hematological and biochemical examination. The blood samples were collected in sterile syringes and were centrifuged. The separated serum was stored at −20°C. The serum concentrations of PTH, 25OHD3, 1,25(OH)2D3 were measured using enzyme-linked immunosorbent assay kits (MBS702033; MBS2600128; MBS736473; mybiosource Inc, San Diego, CA, USA) according to the manufacturer’s instructions. Interleukin-2 (IL-2) and interleukin-6 (IL-6) concentrations were analyzed by using ELISA kits (E-EL-Ch0120; E-EL-Ch0228; Elabscience Inc, Bethesda, MD, USA) following the manufacturer’s instructions. The concentrations of serum Ca and P were measured according to the colorimetric method using a biochemical analyzer (Hitachi modular system, Hitachi Ltd., Tokyo, Japan).

At the end of experiment, six birds were randomly selected from each treatment group and euthanized by dislocation of head. Right legs were immediately collected and refrigerated to determine the mechanical properties of the tibias. Tibia weight was measured by analytical weight balance while thickness was assessed with a Vernier Caliper.

At the end of experiment a total of 54 laying hens were placed in metabolic cages for seven days (six birds per treatment). Excreta samples of each hen were collected and immediately stored at −20°C. During collection, care was taken to avoid contamination from feathers, feed, and foreign materials. Willey It was pulverized with a mill and used for analysis. Nutrient digestibility (%) (Ca and P) was analyzed by the AOAC [12].

### Statistical analysis

The experiment was conducted using a completely randomized design with a factorial structure. The effect of 25OHD3 (0, 25, and 50 μg/kg) and Ca levels (3.5%, 4.0%, and 4.5%) was statistically analyzed using a 3×3 factorial design with a general linear model procedure in SAS version 9.2 (SAS Inst. Inc., Cary, NC, USA). Results were considered significantly different using Duncan multiple range test if their p-values were <0.05.

## RESULTS AND DISCUSSION

### Egg production performance

There was no interactive effect (p>0.05) between 25OHD3 and Ca concentrations for none of the evaluated production parameters of laying hens (Table 2). However, the results showed that egg production, feed intake, and egg weight had significantly increased (p<0.05) in hens that had received 4.5% Ca in their diet, more so than those that had received 3.5% to 4.0% Ca (Table 2). These results support an earlier study by Costa et al [13], who reported that Ca plays an essential role in eggshell formation, and that increases in its dietary level contributed to improved eggshell synthesis and egg production. The improvement in egg production of hens fed 4.5% Ca suggests an increased Ca availability for eggshell formation, in accordance with other researchers in this field [14]. Costa et al [15] tested 3.0% to 5.0% dietary Ca levels and did not find any effect on feed intake. Araújo et al [16] found a lower feed intake when Ca levels were increased from 3.5% to 4.2% in the post-molting period of commercial layers. Our results deviated from these findings. Possibly, this is due to the fact that in the prior research only Ca was added to the diets, whereas in present study Ca was added in combination with VD3 sources. Our resulting Ca dietary requirement estimate of 4.5% in layers is accordingly higher than that reported by Rodrigues et al [17], who recommended a level of 3.5% Ca. Salvador et al [18] obtained a better FCR with 25OHD3. However, in present study the Ca given in combination with cholecalciferol +25OHD3 numerically resulted in improved FCR.

The hens fed 50 μg/kg 25OHD3 in their diet presented with more (p<0.05) egg production than those fed 0, 25 μg/kg 25OHD3 (Table 2). Kakhki et al [19] reported similarly that the addition of 25OHD3 in the diet of laying hens together with basal levels of VD3 increased egg production. The observed positive effects of dietary supplementation with 25OHD3 on egg production might be because of increased villus surface area in the jejunum and duodenum affecting absorption of nutrients from the diet [20]. Additionally, VD3-supplemented hens increase follicle stimulating hormone and luteinizing hormon concentration levels implied that dietary addition of sufficient VD3 induce the feedback effect of hypothalamic and pituitary hormone secretion, and positively affect follicle development [21]. Accordingly, these results could account for the beneficial effects of 25OHD3 supplementation on hens’ performance, possibly related to reproductive hormones.

### Egg shell and tibia bone qualities

Eggshell and tibia bone parameters were neither (p<0.05) influenced by their interaction (Table 3). Dietary Ca levels influenced (p<0.05) eggshell thickness and tibia bone length. In contrast, there was no significant effect (p>0.05) on eggshell quality among the 25OHD3 supplementation groups (Table 3). These results are consistent with those obtained by do Nascimento et al [22], who showed no interaction between levels of Ca (2.85%, 3.65%, 4.45%, and 5.25%) and various VD3 sources (cholecalciferol, 25OHD3, and 1,25(OH)2D3) on eggshell quality. That study confirmed that higher shell thickness when Ca levels in the diet of commercial layers were increased from 2.85% to 5.25%, absent the effects of VD3 sources [22]. Jiang et al [23] also showed that layers fed diet containing 2.62% Ca have a lower eggshell breaking strength than those fed diet with 3.7% or 4.4% Ca, Alternatively, including hens fed a diet supplemented with 25OHD3 had no significant effect on eggshell quality.

Tibia bone length linearly increased (p<0.05) with dietary Ca levels (Table 3). When dietary Ca levels are high, the ultimobranchial glands are stimulated to secrete calcitonin, reducing bone resorption and consequently, increasing bone strength [24,25]. Tibia bone thickness was found to be significantly (p<0.05) higher in group given dietary 25OHD3 supplemental level at 50 μg/kg than in other groups (Table 3). The role of VD3 in Ca metabolism is crucial for bone formation in poultry [19]. Our result may be attributed to the fact that Ca metabolism of birds depend in the activity of cholecalciferol, 25OHD3, and 1,25(OH)2D3 [26]. Sahin et al [27] reported that dietary 25OHD3 supplemental levels at 0 to 500 IU/kg linearly affect bone mineralization of laying quails. Our findings suggested that suitable supplemental dosage of 25OHD3 for bone structure of hens from 61 to 70 weeks of age is 50 μg/kg.

### Serum biochemical parameter concentrations

The influence of dietary concentration of Ca and 25OHD3 on the blood parameters of laying hens is presented in Table 4. The results indicated interaction (p<0.05) between Ca and 25OHD3 occurred the highest PTH in laying hens that received dietary concentration of Ca and 25OHD3 of 3.5% and 50 μg/kg, respectively, or 4.0% and 50 μg/kg, respectively. Stimulation of PTH in the kidneys increase conversion of 25OHD3 to 1,25(OH)2D3 affecting Ca absorption [19]. Our observed interaction effect of dietary Ca and 25OHD3 on the serum PTH concentration in hens could be because of a correlation between VD3 and PTH by Ca and P absorption, regulation of PTH, and bone mineralization [28,29].

Furthermore, the interactive effect (p<0.05) between Ca and 25OHD3 was such that dietary 25OHD3 linearly increased (IL-6) at all Ca levels (Table 4). Interleukin-2 (IL-2) was also linearly increased (p<0.05) in hens that received Ca up to 4.5% while interaction had no significant effects (p>0.05) between Ca and 25OHD3 in the layer diet (Table 4). VD and vitamin D receptors (VDR) have been shown to be important regulators of the immune system [7]. Our results may be related to the study [30] showing that dietary 25OHD3 affect immunoregulation related to VDR and regulatory T cell affecting various immunity cytokines production. Hens fed dietary supplementation with 25OHD3 induce inhibited the growth of pathogens and increased the production of immune cytokines because of increased 1α-hydroxylase and VDR in the liver [7,31].

The concentration of serum Ca was significantly (p<0.05) higher in each group fed 4.5% Ca or 50 μg/kg 25OHD3 than in other groups (Table 4). This finding is similar to those of previous study [23] that included up to 4.4% Ca in the diet of layers, and in which linearly increased Ca concentration was observed in subsequent tests of serum.

No significant difference in the concentration of serum P was observed (p>0.05) between the groups (Table 4). Jiang et al [23], consistent with our observations, showed that layers maintain their concentration of serum P with dietary Ca levels in the 2.62% to 4.4% range.

25OHD3 concentration was higher (p<0.05) in the serum of hens that had 4.5% Ca or 50 μg/kg 25OHD3 included in their diets than in those that were offered alternative diets, but its active form (1,25(OH)2D3) was not influenced by dietary supplementation of Ca or 25OHD3 (Table 4). These results were consistent with the prior study of Kappeli et al [32], where the researchers showed that hens fed VD3 supplemented as 25OHD3 (37.5 μg/kg) higher 25OHD3 concentration on the blood.

### Nutrition digestibility of Ca and P

The nutrition digestibility of Ca and P was unaffected (p>0.05) by an interactive effect of dietary concentrations between Ca and 25OHD3 (Table 5).

Ca digestibility was similar (p>0.05) in hens fed dietary Ca levels of 3.5% to 4.5% (Table 5). Swiatkiewicz et al [33] found that ash digestibility had a similar result among hens supplemented with dietary Ca concentrations (3.2% to 4.2%) and total amount of excreted and retained Ca increased linearly with an increasing dietary Ca levels. Pelicia et al [34] supplemented layer diets with 3.0% to 4.5% Ca, and observed that the highest dietary Ca level was correlated with the highest blood Ca concentration and highest eggshell percentage, suggesting an association between dietary Ca and Ca deposits at second cycle layers.

Ca digestibility significantly increased (p<0.05) over the cholecalciferol group in groups fed a diet supplemented with 25 and 50 μg/kg of 25OHD3 (Table 5). Cholecalciferol is fat-soluble and its absorption is facilitated by bile salts, while 25OHD3 has a highly polar molecular structure and is less affected by bile salts [35]. Accordingly, more 25OHD3 is absorbed (1.5×) than the same level of cholecalciferol (2,760 IU/kg) in the bird’s intestine and this affects calcium absorption and metabolism in broilers [5]. Koreleski and Swiątkiewicz [36] verified that hens fed 25% to 75% 25OHD3 of the total VD3 sources showed higher relative Ca concentration in eggshells, and determined that this may be linked to more Ca absorption [19].

P digestibility linearly increased (p<0.05) as dietary Ca level increased from 3.5% to 4.5%, while the tested dietary 25OHD3 levels did not affect (p>0.05) P digestibility in hens (Table 5). The ions of Ca and P were predominantly absorbed in the upper portion (duodenum and upper jejunum) of the small intestine, and then deposited in the form of calcium phosphate (Ca3(PO4)2). Bar et al [37] mentioned that the correlation between Ca and P in layer diets affected Ca and P absorption in which optimal ratio was presented Ca:AP = around 11 to 13:1. On the basis of these results, the Ca requirement for aged hens (450 to 650 days) should be slightly higher than those provided for by the NRC. These results were consistent with our finding that P digestibility increased in 61-to-70-week-old s hens with higher dietary Ca levels (4.0% to 4.5%).

## CONCLUSION

Our results showed the interactive effect between Ca (3.5%, 4.0%, and 4.5%) and 25OHD3 (0, 25, and 50 μg/kg) on serum PTH and IL-6 of laying hens. In addition, dietary supplementation of 25OHD3 improved egg production, tibia bone thickness, and Ca digestibility of up to 50 μg/kg in laying hens aged 61 to 70 weeks. Thus, diet supplemented with 4.5% Ca in combination with 50 μg/kg 25OHD3 could be used during finishing stage period of laying hens.

## Figures and Tables

**Table 1:** Basal diet composition for different treatments

Items	Ca level (%) + vitamin D_3_ (3,000 IU/kg)		

	3.5	4.0	4.5
Ingredients (%)			
Corn	57.4	57.5	57.7
Wheat bran	8.70	6.60	4.45
Soybean meal	23.3	23.9	24.5
Corn-DDGS	1.00	1.00	1.00
Limestone	8.50	9.80	11.1
DCP	0.800	0.850	0.900
Mineral premix^1)^	0.100	0.100	0.100
Vitamin premix^2)^	0.100	0.100	0.100
Methionine (99%)	0.150	0.150	0.150
Total	------------------------- 100 ----------------------		
Calculated chemical composition			
Metabolic energy (kcal/kg)	2,800	2,800	2,800
Crude protein (%)	16.0	16.0	16.0
Calcium (%)	3.50	4.00	4.50
Total protein (%)	0.500	0.500	0.500
Available phosphorus (%)	0.350	0.350	0.350
Total methionine (%)	0.400	0.400	0.400
Total cysteine (%)	0.270	0.270	0.270
Total lysine (%)	0.830	0.830	0.830

1) Mineral supplemented provided per kilogram of diet: Fe, 60 mg, Cu,10 mg; Zn 80 mg; Mn, 110 mg; Iodine, 0.48 mg; Se, 0.40 mg.

2) Vitamin supplement provided per kilogram of diet: vitamin A, 10,000 IU; vitamin E, 30 IU; vitamin B_1_, 3 mg; vitamin B_2_, 7 mg; vitamin B_6_, 0.15 mg; vitamin B_12_, 0.025 mg; vitamin K_3_, 3 mg; Niacin, 50 mg; vitamin C, 200 mg; Pantothenic acid, 12 mg; choline, 500 mg; biotin, 0.15 mg; folic acid, 1.5 mg. Vitamins D_3_ was included at the expense of inert material at 3,000 IU/kg at the following three levels (per kg) of cholecalciferol+25OHD3: 600 mg+0 μg, 400 mg+25 μg, and 200 mg+50 μg, respectively.

**Table 2:** Production performance of laying hens fed dietary concentrations of Ca and 25OHD3 from 61 to 70 weeks of age

Main effect	Egg production (%)	Feed intake (g/d)	Egg weight (g)	Feed conversion ratio (g/g)
Ca (%)				
3.5	76.8^b^	112^c^	62.9^b^	2.33
4.0	77.7^ab^	114^b^	62.4^b^	2.35
4.5	78.2^a^	115^a^	63.7^a^	2.31
25OHD3 (μg/kg)				
0	76.7^b^	114	63.3	2.34
25	77.4^ab^	114	62.9	2.33
50	78.6^a^	114	62.8	2.31
	------------------------- Probability >F ------------------------			
Source of variation				
Ca	0.05	<0.01	0.01	0.24
25OHD3	0.01	0.40	0.54	0.32
Ca×25OHD3	0.84	0.96	0.97	0.97

25OHD3, 25-hydroxy vitamin D3.

a–c Means within a column with unlike superscripts differ significantly (p<0.05).

**Table 3:** Egg shell and tibia bone qualities from laying hens fed dietary concentrations of Ca and 25OHD3

Main effect	Egg shell		Tibia bone		
	
	Weight (g)	Thickness (mm)	Length (cm)	Thickness (mm)	Weight (g)
Ca (%)					
3.5	9.54	0.352^b^	11.7^b^	8.46	13.9
4.0	9.76	0.364^b^	12.0^a^	8.46	14.0
4.5	9.77	0.384^a^	12.2^a^	8.61	14.5
25OHD3 (μg/kg)					
0	9.67	0.364	11.9	8.46^ab^	14.1
25	9.58	0.377	12.0	8.37^b^	14.1
50	9.83	0.369	12.1	8.69^a^	14.2
	------------------------------------------------------------------------------- Probability >F ---------------------------------------------------------------------------				
Source of variation					
Ca	0.62	<0.01	0.05	0.44	0.40
25OHD3	0.63	0.71	0.32	0.04	0.96
Ca×25OHD3	0.72	0.68	0.14	0.84	0.51

25OHD3, 25-hydroxy vitamin D3.

a,b Means within a column with unlike superscripts differ significantly (p<0.05).

**Table 4:** Serum biochemical concentration of laying hens fed dietary concentrations of Ca and 25OHD3

Main effect	PTH (pg/mL)	IL-2 (mg/dL)	IL-6 (mg/dL)	Ca (mg/dL)	P (mg/dL)	25OHD3 (nmol/L)	1,25(OH)2D3 (nmol/L)
Ca (%)							
3.5	133	13.9^b^	45.4^c^	26.9^b^	4.87	14.5^b^	14.1
4.0	132	15.7^ab^	51.7^b^	26.0^b^	5.00	14.3^b^	14.9
4.5	129	19.8^a^	56.3^a^	28.0^a^	4.88	15.9^a^	15.3
25OHD3 (μg/kg)							
0	130	17.1	40.0^b^	26.2^b^	4.79	14.1^b^	14.3
25	131	15.3	50.1^b^	26.5^b^	4.97	14.8^ab^	14.8
50	133	16.9	55.3^a^	28.2^a^	5.00	15.9^a^	15.2
	------------------------------------------------------------------------------------ Probability >F --------------------------------------------------------------------------						
Source of variation							
Ca	0.44	0.04	<0.01	0.05	0.86	0.02	0.68
25OHD3	0.62	0.69	0.01	0.04	0.69	0.04	0.82
Ca×25OHD3	0.05	0.33	0.04	0.29	0.91	0.09	0.42

25OHD3, 25-hydroxy vitamin D3; PTH, parathyroid hormone; IL, interleukin-2; 1,25(OH)2D3, 1,25-dihydroxycholecalciferol.

a–c Means within a column with unlike superscripts differ significantly (p<0.05).

**Table 5:** Nutrition digestibility of Ca and P from laying hens offered dietary concentrations of Ca and 25OHD3

Main effect	Ca (%)	P (%)
Ca (%)		
3.5	59.4	52.5^b^
4.0	61.8	55.2^ab^
4.5	62.0	56.8^a^
25OHD3 (μg/kg)		
0	58.8^b^	53.8
25	61.7^a^	54.7
50	62.7^a^	56.0
	Probability >F	
Source of variation		
Ca	0.38	0.01
25OHD3	<0.01	0.78
Ca×25OHD3	0.09	0.54

25OHD3, 25-hydroxy vitamin D3.

a,b Means within a column with unlike superscripts differ significantly (p<0.05).
